# Environmental Persistence of *Bacillus anthracis* and *Bacillus subtilis* Spores

**DOI:** 10.1371/journal.pone.0138083

**Published:** 2015-09-15

**Authors:** Joseph P. Wood, Kathryn M. Meyer, Thomas J. Kelly, Young W. Choi, James V. Rogers, Karen B. Riggs, Zachary J. Willenberg

**Affiliations:** 1 United States Environmental Protection Agency, Office of Research and Development, National Homeland Security Research Center, Research Triangle Park, North Carolina, United States of America; 2 Oak Ridge Institute for Science and Education, Research Triangle Park, NC, United States of America; 3 Battelle Memorial Institute, Columbus, Ohio, United States of America; Loyola University Chicago, UNITED STATES

## Abstract

There is a lack of data for how the viability of biological agents may degrade over time in different environments. In this study, experiments were conducted to determine the persistence of *Bacillus anthracis* and *Bacillus subtilis* spores on outdoor materials with and without exposure to simulated sunlight, using ultraviolet (UV)-A/B radiation. Spores were inoculated onto glass, wood, concrete, and topsoil and recovered after periods of 2, 14, 28, and 56 days. Recovery and inactivation kinetics for the two species were assessed for each surface material and UV exposure condition. Results suggest that with exposure to UV, decay of spore viability for both *Bacillus* species occurs in two phases, with an initial rapid decay, followed by a slower inactivation period. The exception was with topsoil, in which there was minimal loss of spore viability in soil over 56 days, with or without UV exposure. The greatest loss in viable spore recovery occurred on glass with UV exposure, with nearly a four log_10_ reduction after just two days. In most cases, *B*. *subtilis* had a slower rate of decay than *B*. *anthracis*, although less *B*. *subtilis* was recovered initially.

## Introduction

Following the intentional release of virulent *Bacillus anthracis* spores via the United States Postal Service in 2001 [1], a need for effective remediation methods has emerged. Inactivation of biological agents through natural attenuation processes may be a potential option for remediation of contaminated outdoor areas following a wide area bioterrorism event, depending on the type of microorganism and environmental conditions. For example, bacterial spores are most resistant to viability decay in the environment, whereas vegetative bacteria are least resistant, and the sensitivity of viruses to environmental conditions is intermediate [2]. Further, since ultraviolet radiation (UV) in sunlight is bactericidal [3], exposure to solar radiation could limit bacterial spore (such as those of *B*. *anthracis*) persistence in the environment [4]. However, there is a lack of data and a need for predictive understanding of biological agent viability in different environments [2].

Historically, most laboratory studies of *Bacillus* spore photochemistry and resistance mechanisms have used 254 nm UV radiation (UV-C) [5–9], as this wavelength was predominantly tested because of its germicidal properties [10,11]. However, natural solar radiation, or sunlight, is considerably more complex than this monochromatic 254 nm UV wavelength. Solar radiation is a mixture of UV, visible, and infrared radiation, with only those UV wavelengths longer than 290 nm reaching the Earth’s surface [4]. That is, solar UV radiation reaching the Earth’s surface is comprised of both UV-A (380–320 nm) and UV-B (320–290 nm) regions, with no UV-C (<290 nm) [4]. Given that UV-B is more environmentally relevant than UV-C, and DNA damage occurs directly through the absorption of energy from solar radiation [4,11], more recent studies have focused on bacterial spore resistance to solar radiation, using either natural sunlight or exposure in a laboratory setting to solar radiation components UV-A and/or UV-B [5,6,12–15]. However, because these studies used *Bacillus subtilis* spores, the relevance of the resulting data to virulent *B*. *anthracis* spores is unknown.

The primary objective of this study was to investigate the effect of simulated sunlight (UV-A/B) on the inactivation kinetics of virulent *B*. *anthracis* Ames spores and *B*. *subtilis* spores, a potential avirulent surrogate. Although a few primary studies and reviews have examined the inactivation of dried *Bacillus* spores on surfaces due to UV [7,16, 17], none have used a virulent *B*. *anthracis* strain in combination with natural or simulated sunlight. Further, the present study used realistic materials that are commonly found outside and exposed to natural sunlight. (Most biological agent persistence studies fail to account for the effect of materials or environmental matrices.) Previous results suggest that a material’s pore structure and texture may shield and provide physical protection to the spores from UV radiation [7,16]. Therefore the current study used *Bacillus* spores dried onto both non-porous and porous materials, to determine their resistance to simulated sunlight.

## Materials and Methods

### Bacterial Spore Preparation

Spores of the virulent *B*. *anthracis* Ames strain were prepared by fermentation as previously described [18]. A culture of *B*. *anthracis* Ames was grown in nutrient broth (BD Diagnostic Systems, Sparks, MD) for 16–18 hours at 37°C on an orbital shaker at 150–200 RPM and used to inoculate a scale-up nutrient broth culture grown for 6–8 hours at 37°C while shaking at 150–200 RPM. This scale-up culture was used to inoculate Leighton-Doi Broth (BD Diagnostic Systems) in a BioFlo 3000 fermentor (New Brunswick Scientific Co., Inc., Edison, NJ) that was operated for approximately 24 hours at 37°C. The culture was harvested and centrifuged at approximately 10,000–12,000 × *g* for 15–20 min at 2–8°C and the pellet was washed twice and resuspended in ice-cold, sterile water. The preparation was heat-shocked at 60°C for 45–60 min, centrifuged, washed twice, and resuspended in ice-cold, sterile water. This suspension was centrifuged at 9,000 × *g* for 2 hours at 2–8°C through an ice-cold, sterile Hypaque-76 (Nycomed Amersham, Princeton, NJ, USA) gradient and the pellet was washed and resuspended in ice-cold, sterile water. Preparations having >95% refractile spores with <5% cellular debris were enumerated, diluted to approximately 1.0 × 10^9^ colony-forming units (CFU)/mL, and stored at 2–8°C.

Spores of *B*. *subtilis* (ATCC 19659; Manassas, VA) were prepared according to AOAC Official Method 966.04, Method II [19] in which *B*. *subtilis* was cultured for 12–14 days on amended nutrient agar. Following incubation, spores were harvested in sterile water and evaluated by phase-contrast microscopy. Preparations having >95% refractile spores with <5% cellular debris were enumerated, diluted to approximately 1.0 × 10^9^ CFU/ml, and stored at 2–8°C.

### Preparation and Inoculation of Material Coupons

Wood (untreated pine lumber, Kingswood Lumber Co.; Columbus, OH) and glass (C1036, Brooks Brothers; Columbus, OH) were cut into 1.9 cm x 7.5 cm coupons; unpainted concrete (5 parts sand, 2 parts cement, Wysong Concrete Products; Fairfield, OH) was poured into a mold to produce coupons 1.9 cm x 7.5 cm, rather than being cut to size. Topsoil (batch # PY1A0597, Gardenscape, Inc.; Columbus, OH) “coupons” were prepared by completely filling (but not compacting) a Parafilm^®^-lined (Pechiney Plastic Packaging Co.; Chicago, IL), 3.5 cm diameter by 1 cm deep Petri dish. (No water was added to the topsoil, and soil moisture was not measured.) Glass and unpainted concrete coupons were sterilized prior to use by autoclaving, and bare wood coupons were sterilized by gamma irradiation using a dose of 40 kilogray. Topsoil ‘coupons’ were not autoclaved or irradiated before use to avoid potentially modifying or altering the physical or chemical characteristics of the soil matrix. Instead, native vegetative microorganisms were suppressed by a post-extraction heat shock treatment for one hour at 65°C, consistent with Brown et al. [20].

Coupons were inoculated with either *B*. *subtilis* or *B*. *anthracis* spores at approximately 1 x 10^8^ CFU per coupon. This inoculation level is comparable to the aforementioned studies investigating the attenuation of spores via UV-A or UV/B, which typically ranged from 10^6^ to 10^7^ CFU per slide [5, 6, 12–14], and is also consistent with other related decontamination studies [18]. Inoculation was accomplished by dispensing a 100 μL aliquot of approximately 1 x 10^9^ CFU/mL as 10 droplets of 10 μL each across the surface of the test coupon. Following inoculation, test coupons were left to dry overnight in the biosafety cabinet. Five coupons of each material were used for the UV-exposed and unexposed test conditions for each experimental time point. One blank coupon of each material was also included with the exposed coupons and with the unexposed coupons for each time point. For testing, the coupons were arranged in five separate positions in an hourglass pattern on the support trays. One exposed (or unexposed) coupon of each of the four materials was placed at each of these five positions to ensure that all coupon materials were equally distributed across the metal support trays. A blank coupon of one of each of the four materials was also placed at each of the outer positions to ensure similar distribution.

### Test Chamber

**Fig 1:**
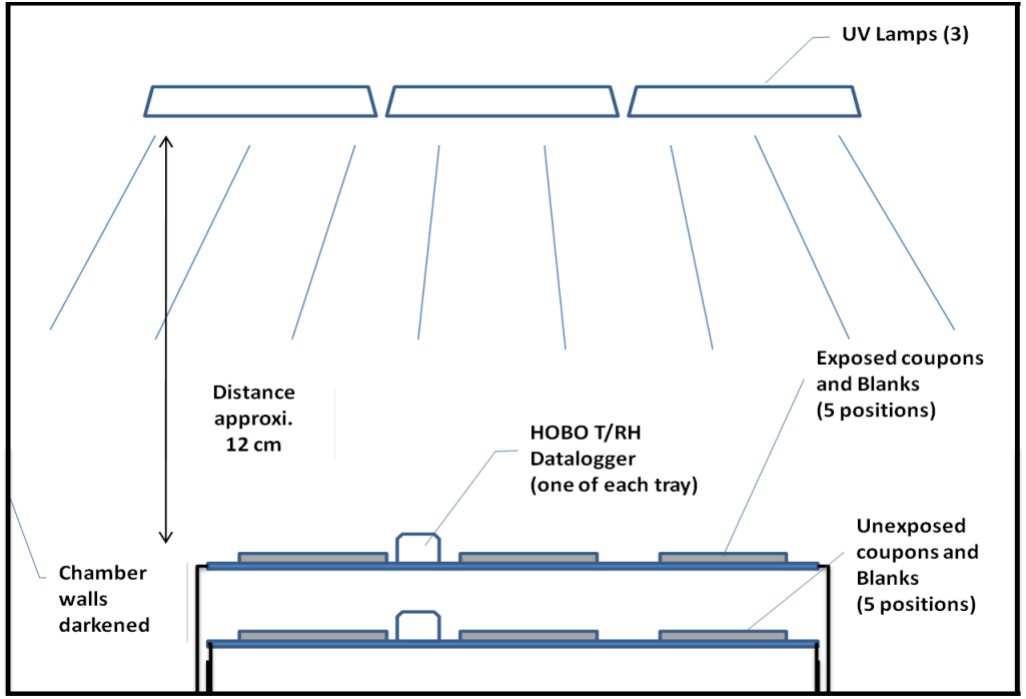
Test chamber schematic (not to scale). Exposed coupons were positioned in a single layer on top of the tray (lined with kraft paper to block UV radiation from hitting lower tray) with unexposed coupons positioned in a single layer underneath the tray supporting the test coupons. Unexposed coupons were also shielded from UV light exposure from the side.

An acrylic compact glove box model 830-ABC (Plas Labs, Inc.; Lansing, MI) exposure chamber was used for testing. The internal dimensions of the chamber were 71 cm (w) x 59 cm (d) x 74 cm (h). ReptiSun^®^ 10.0 Linear Fluorescent UV-B lamps (15 Watts, 48 cm long, Zoo Med Laboratories; San Luis Obispo, CA) were positioned inside the top of the chamber to provide both UV-A and UV-B radiation to which the coupons were exposed. The chamber was also covered with black paper to shield the interior from external light and minimize reflected UV-A/B radiation. Two sets of coupons were used: those exposed to UV radiation (exposed) and those shielded from UV radiation (unexposed). Exposed coupons (N = 5/material) and their associated blank coupons (N = 1/material) were placed onto raised support trays about 12 cm below the UV lamps. Unexposed coupons (N = 5/material) and associated blank coupons (N = 1/material) were placed on a second support tray, beneath the test coupon tray, which was lined with kraft paper to shield coupons below from direct UV-A/B radiation. UV radiation was measured with commercially available Solarmeter^®^ Digital Ultraviolet radiometers, Model 5.7 (total UV), Model 6.2 (UV-B), and Model 8.0 (UV-C) (Solar Tech, Inc.; Harrison Township, MI). UV-A and UV-B intensity were monitored at five locations within the exposed coupon array and five corresponding locations in the unexposed coupon array (UV-A was determined as the difference between measured total UV and UV-B). The UV intensities found at the five different positions in the test coupon arrays typically ranged from about 5% less than to 10% greater than the average UV intensity over all five positions. All UV exposure testing began at ambient temperature and relative humidity (RH), approximately 22°C and 50% RH. A HOBO U10 data logger (Onset Computer Corp.; Bourne, MA) was used to monitor temperature and RH continuously and measure exposure time during testing. Temperature and RH were monitored (not controlled) both near the coupons exposed to UV radiation and near the unexposed coupons shielded from UV radiation. Fig 1 shows a schematic representation of the test chamber, including the positions of the coupons relative to the UV lamps Additional details on the UV-A/B test chamber and methods may be found elsewhere [21].

### UV Exposure Conditions

On the day following spore inoculation, the coupons were transferred to the test chamber and arranged in the aforementioned hourglass pattern, ensuring five inoculated coupons and one blank coupon for UV-A/B exposure, and five inoculated coupons and one blank for non-exposure for each experiment performed. Four separate experiments were performed for each microorganism, using elapsed times of 2, 14, 28, and 56 days. The UV-A/B exposure occurred using alternating cycles of 12 hours of UV-A/B exposure (lamps on) and 12 hours of darkness (lamps off), to simulate diurnal conditions. (For example, in the 14-day test, the total UV-A/B exposure period was 168 hours.) Exposure periods were performed sequentially as separate experiments and were not simultaneous or overlapping.

The spectrum and intensity of UV radiation at the Earth’s surface is highly variable and is affected by a number of factors, such as time of day, day of year, geographical location, altitude, atmospheric pollution, stratospheric ozone column, and cloud cover. Peak summertime UV-B levels at the earth’s surface, as reported in a few studies, ranged from 40 to 150 *μ*W/cm^2^ [22–24]; therefore a target level of 70 *μ*W/cm^2^ UV-B was selected for the present study. With 12 hours exposure per day, this intensity of UV-B equates to a daily dose of approximately 3.0 J/cm^2^ (or 30 kJ/m^2^), a level similar to the daily UV-B dose received during the summer months in Raleigh, NC [25]. Because the adverse effects of solar radiation on biological systems are mostly attributed to UV-B [4, 26], no UV-A level was specifically targeted, but UV-A was monitored throughout all experiments to ensure a consistent level (∼100 *μ*W/cm^2^). (Average UV-A levels in Raleigh, NC, ranged from 300–2000 *μ*W/cm^2^ during the summer months of 2014 [25]). The target UV-C exposure level for tests was 0 *μ*W/cm^2^, as UV-C does not reach the earth’s surface [27].

### Sample Extraction and Spore Recovery

Following the persistence test period, coupons (both UV-exposed and non-exposed) were aseptically transferred to 50 mL conical tubes containing 10 mL of phosphate buffered saline (PBS) with 0.1% Triton^®^ X-100. With the exception of bare concrete, the coupons were extracted by agitation on an orbital shaker (Environ Shaker model 3827, Lab-Line Instruments, Thermo Scientific; Pittsburgh, PA) for 15 minutes at 200 RPM at room temperature. For the bare concrete, instead of the agitation period, coupons were sonicated (Model 1200, Branson Ultrasonics; Danbury, CT) for 45 minutes. The use of topsoil as a coupon required an additional heat shock procedure to minimize interference by native microorganisms. After the topsoil was extracted in the PBS/Triton^®^ X-100 solution, the recovered supernatant was heat-shocked in a water bath (Precision Model 280; Thermo Scientific) at 65°C for one hour. For all coupon extracts, sequential 10-fold serial dilutions were prepared in sterile filtered water (SFW) and subsequently spread-plated in triplicate onto tryptic soy agar (TSA) plates. Plates were incubated at 35 ± 2°C for 18–24 hours, and CFU were enumerated by visual inspection of the plates. The inoculum for each species was titered on the day of coupon inoculation in each experiment.

### Spore Confirmation

To examine the possibility of contamination from native microorganisms in the topsoil coupons during the two-day test, additional tests were performed. These tests included a visual confirmation of colony morphology and the use of polymerase chain reaction (PCR). For the PCR confirmation, the topsoil extracts for both *B*. *anthracis* and *B*. *subtilis* were replated onto tryptic soy agar and incubated at 35 ± 2°C for 18–24 hours. For each spore species, 50 individual colonies were selected with a 1 μL disposable sterile loop and suspended in 200 μL of 10 mmol/L Tris-HCl (pH 8) in a 1.5 mL microcentrifuge tube containing a 0.22 μM filter unit (Millipore; Bedford, MA). The tubes were then heated at 95°C for 20 minutes and centrifuged (Avanti J-26XPI, Beckman Coulter, Inc.; Brea, CA) at 6,000 x *g* for two minutes. The bacterial lysate was collected and used in a PCR assay to confirm the respective organisms (*B*. *anthracis* Cap B domain FAM primers—proprietary sequences, P/N 121P01, Invitrogen Corp.; Carlsbad, CA; *B*. *subtilis* RopB primers—forward 5’-GAT GTT GTT TAT GTC CGC ATT GA-3’, reverse 5’-GAG CAC GCA AAA GAA CCG TAA-3’, Taqman-MGB probe 5’-CAC ACG TAA GTT GCC G-3’).

### Numerical and Statistical Analysis

To establish inactivation kinetics, the surviving fraction of *B*. *anthracis* and *B*. *subtilis* spore population after each elapsed time period was determined from the quotient N_t_/N_0_, in which *N*_*t*_ was the average number of CFU after the elapsed time period and *N*_*0*_ was the initial population (the inoculum amount) of spores. Inactivation data that exhibited exponential decay over time were fitted to a log_10_-based first order inactivation model [28]:

$$\frac{{\text{N}}_{\text{t}}}{{\text{N}}_{0}}={\text{e}}^{\text{-kt}}$$ (1)

In this equation, *k* is the inactivation constant, the slope of the linear portion of the curve when the data are plotted in log form, and *t* is the duration of the test (in days). Survival curves were constructed by plotting the quotient N_t_/N_0_ on a log scale versus elapsed time for both the UV-A/B exposed materials as well as the materials shielded (unexposed) from UV exposure. A lower value for N_t_/N_0_ indicates a higher sensitivity to inactivation by UV radiation.

Due to the biphasic nature of the survival curves [29], spore decay was quantified and plotted in two different phases. The first phase represents the initial, rapid decay seen from Day 0 to Day 2, and would include the inability to physically recover the spores from the materials, as well as loss due to inactivation from UV exposure. The second phase represents the slower inactivation seen from Day 2 to Day 56. For the initial phase, most comparisons of N_t_/N_0_ were made using the Satterthwaite *t*-test to account for unequal variances (TTEST in SAS version 9.3 software). Pooled variance *t*-tests were used for these comparisons: on wood to compare UV-exposed versus unexposed spores and to compare both *Bacillus* spore species; to compare unexposed *B*. *anthracis* spores on concrete versus glass, concrete versus soil, and glass versus soil; to compare *B*. *subtilis* UV-exposed spores on concrete versus glass; and to compare concrete versus wood materials for the UV-exposed *B*. *anthracis* and unexposed *B*. *subtilis* spore UV combinations. P-values were calculated based on these statistical tests, with differences in spore recovery determined to be significant using a 5% level (p-value < 0.05).

For the second phase of decay, the data were first log-transformed so that the estimated slopes represent exponential decay rates. We then compared the 95% confidence intervals for the slopes of regression equations for the various experimental conditions to determine if there were significant differences. Decay rates were calculated in units of day^-1^. All regressions and statistical analyses were performed using SAS version 9.3 software (SAS Institute Inc; Cary, NC) and figures were made in SigmaPlot 12.3 (Systat Software Inc.; San Jose, CA).

## Results and Discussion

### Environmental Conditions

The UV intensities measured at each of the five positions for the duration of each test confirmed average UV-A/B levels for the exposed coupons to be approximately 102 μW cm^2^ UV-A and 69 μW/cm^2^ UV-B (Table 1), with no detectable UV-C (<1 μW/cm^2^). There was no detectable UV-A, UV-B, or UV-C for the unexposed coupons (data not shown). Using 12 hours exposure per day, total UV-B doses corresponding to the 2, 14, 28, and 56 day tests were 57.8, 431.8, 857.6, and 1683.8 kJ/m^2^ for *B*. *anthracis* and 57.8, 419.1, 839.5, and 1693.4 kJ/m^2^ for *B*. *subtilis*, respectively. The UV-A doses were 88.1, 641.1, 1270.1, and 2443.4 kJ/m^2^ for *B*. *anthracis* and 87.3, 589.7, 1245.9, and 2443.4 kJ/m^2^ for *B*. *subtilis*, for the 2, 14, 28, and 56 day test periods, respectively.

**Table 1:** Summary of environmental conditions for each experiment.

Test Condition	2 days	2 days	14 days	14 days	28 days	28 days	56 days	56 days
	*Bacillus anthracis*	*Bacillus subtilis*	*B*.*a*.	*B*.*s*.	*B*.*a*.	*B*.*s*.	*B*.*a*.	*B*.*s*.
UV-A^§^ Average ± SD	102 ± 4.3	101 ± 3.5	106 ± 7.7	97.5 ± 4.0	105 ± 7.1	103 ± 6.6	101 ± 8.1	101 ± 8.5
UV-B^§^ Average ± SD	67.0 ± 4.7	66.9 ± 4.5	71.4 ± 3.3	69.3 ± 3.7	70.9 ± 3.8	69.4 ± 3.7	69.6 ± 4.4	70.0 ± 3.9
**UV ON** ^α^								
Exposed—Temp Average ± SD	28.3 ± 1.4	28.3 ± 1.3	26.3 ± 2.8	25.0 ± 0.8	26.6 ± 1.0	26.8 ± 1.0	28.4 ± 1.1	29.0 ± 1.1
Exposed—RH Average ± SD	31.3 ± 3.6	33.0 ± 2.9	46.2 ± 5.4	37.6 ± 2.3	35.4 ± 2.7	38.4 ± 2.9	32.0 ± 3.0	33.2 ± 7.0
Unexposed-Temp Average ± SD	25.0 ± 0.7	25.0 ± 0.7	23.6 ± 1.6	22.4 ± 0.4	22.6 ± 0.5	23.2 ± 0.7	24.4 ± 0.6	24.8 ± 0.8
Unexposed—RH Average ± SD	41.1 ± 4.6	42.1 ± 2.5	57.7 ± 5.1	45.1 ± 2.5	47.1 ± 1.8	46.5 ± 2.6	41.0 ± 2.9	33.2 ± 7.0
**UV OFF** ^α^								
Exposed—Temp Average ± SD	23.8 ± 1.0	23.8 ± 1.0	21.6 ± 1.1	21.3 ± 0.5	21.8 ± 0.9	21.6 ± 1.1	22.8 ± 0.9	23.1 ± 0.9
Exposed—RH Average ± SD	32.5 ± 0.6	36.2 ± 1.0	50.9 ± 3.5	44.2 ± 2.2	45.9 ± 2.8	46.7 ± 3.1	41.4 ± 3.3	34.1 ± 6.4
Unexposed—Temp Average ± SD	23.4 ± 0.5	23.3 ± 0.5	21.4 ± 0.9	21.3 ± 0.4	21.7 ± 0.5	21.5 ± 0.8	22.6 ± 0.6	23.0 ± 0.6
Unexposed—RH Average ± SD	36.5 ± 0.8	40.4 ± 2.0	56.3 ± 4.2	47.6 ± 2.3	49.9 ± 1.6	50.9 ± 2.3	44.6 ± 3.0	36.2 ± 7.3

^§^All UV intensity entries are reported in μW/cm^2^.

^α^UV ON and UV OFF refer to diurnal 12-h periods of alternating illumination and darkness in test chamber; averages shown are over all periods in the indicated elapsed time period. Temp = temperature in degrees Celsius, RH = percent relative humidity, SD = standard deviation.

When the UV-A/B lights were on, temperature and RH at the unexposed coupons were within approximately 3°C and within 10% RH, respectively, of the temperature and RH of the exposed coupons (27°C, 35% RH; Table 1). When the UV lights were off the two sets of coupons were within 0.5°C and 5% RH (22°C, 41% RH; Table 1).

### Overall Spore Persistence Results

**Fig 2:**
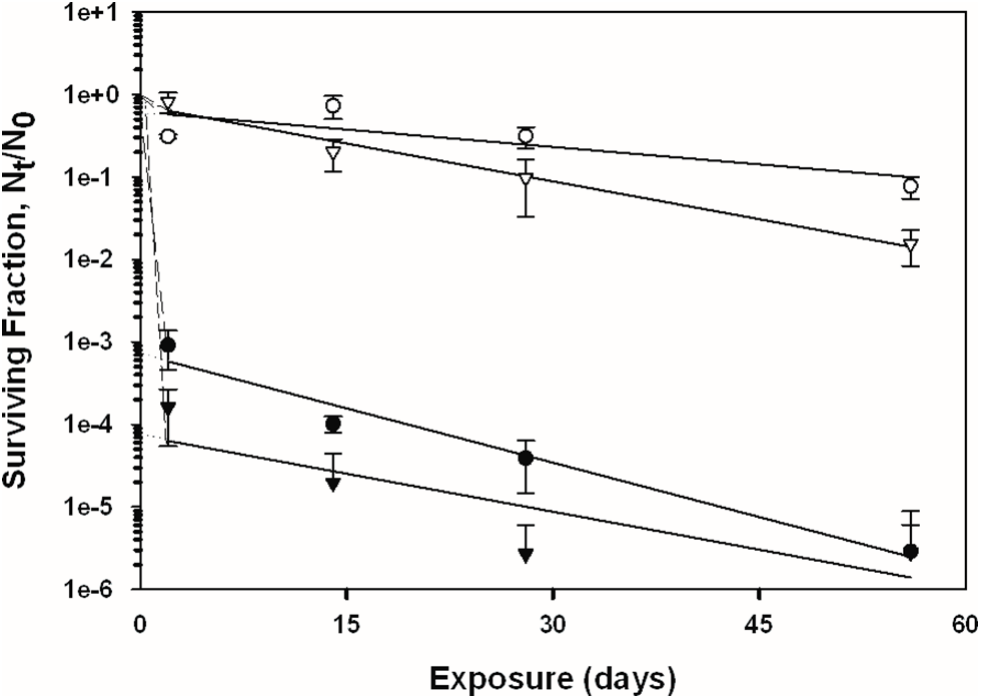
Inactivation curves of *B*. *subtilis* and *B*. *anthracis* spores on glass. Circles represent *B*. *subtilis*, triangles represent *B*. *anthracis*, shaded symbols represent coupons exposed to UV, and non-shaded symbols represent the coupons that were not exposed to UV. The data points and error bars are the averages and standard deviations of results from five replicate coupons. The surviving fraction of spores after each experiment was calculated by N_t_/N_0_, where the viable spore titer at any given time point (*N*_*t*_) was divided by the initial spore titer (*N*_*0*_). Dashed and solid lines represent the initial (first phase) and final (second phase) slopes of the curve, respectively.

**Fig 3:**
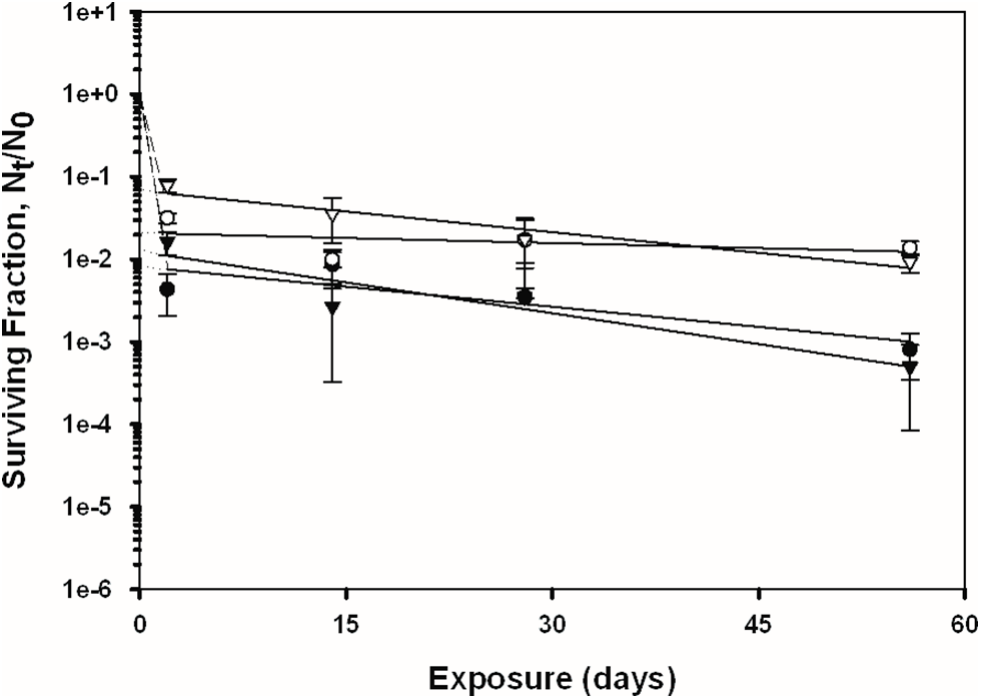
Inactivation curves of *B*. *subtilis* and *B*. *anthracis* spores on bare pine wood. Circles represent *B*. *subtilis*, triangles represent *B*. *anthracis*, shaded symbols represent coupons exposed to UV, and non-shaded symbols represent the coupons that were not exposed to UV. The data points and error bars are the averages and standard deviations of results from five replicate coupons. The surviving fraction of spores after each experiment was calculated by N_t_/N_0_, where the viable spore titer at any given time point (*N*_*t*_) was divided by the initial spore titer (*N*_*0*_). Dashed and solid lines represent the initial (first phase) and final (second phase) slopes of the curve, respectively.

**Fig 4:**
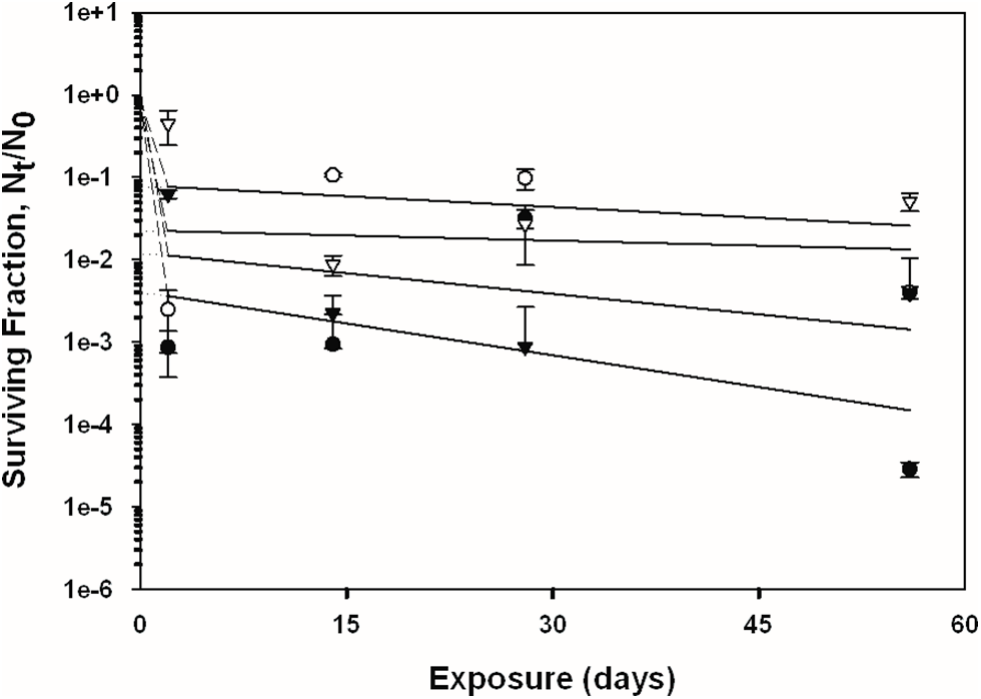
Inactivation curves of *B*. *subtilis* and *B*. *anthracis* spores on unpainted concrete. Circles represent *B*. *subtilis*, triangles represent *B*. *anthracis*, shaded symbols represent coupons exposed to UV, and non-shaded symbols represent the coupons that were not exposed to UV. The data points and error bars are the averages and standard deviations of results from five replicate coupons. The surviving fraction of spores after each experiment was calculated by N_t_/N_0_, where the viable spore titer at any given time point (*N*_*t*_) was divided by the initial spore titer (*N*_*0*_). Dashed and solid lines represent the initial (first phase) and final (second phase) slopes of the curve, respectively.

**Fig 5:**
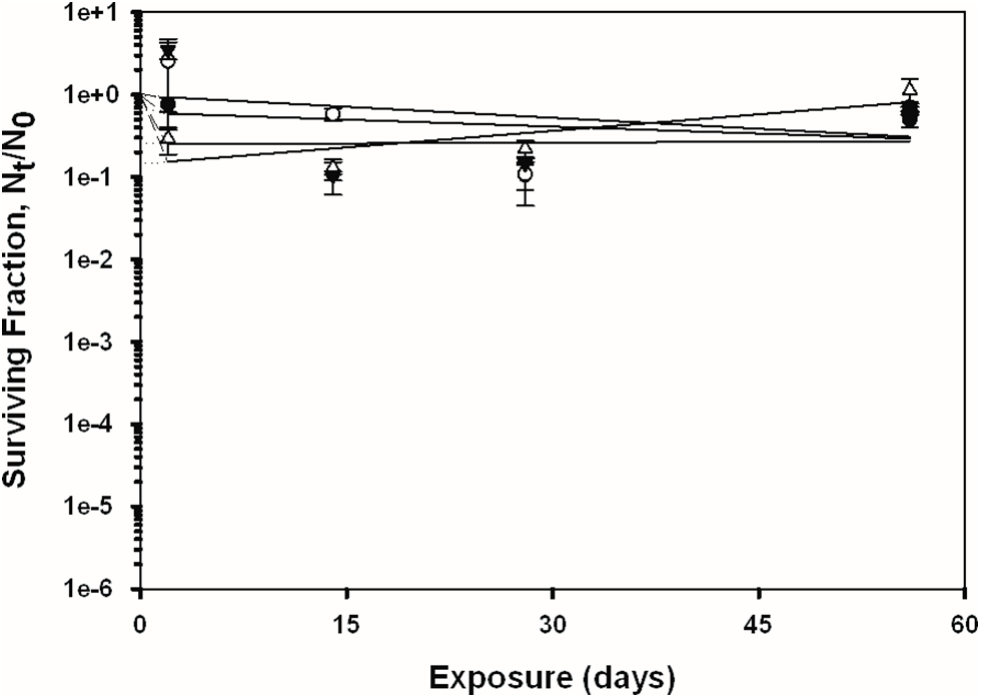
Inactivation curves of *B*. *subtilis* and *B*. *anthracis* spores on topsoil. Circles represent *B*. *subtilis*, triangles represent *B*. *anthracis*, shaded symbols represent coupons exposed to UV, and non-shaded symbols represent the coupons that were not exposed to UV. The data points and error bars are the averages and standard deviations of results from five replicate coupons. The surviving fraction of spores after each experiment was calculated by N_t_/N_0_, where the viable spore titer at any given time point (*N*_*t*_) was divided by the initial spore titer (*N*_*0*_). Dashed and solid lines represent the initial (first phase) and final (second phase) slopes of the curve, respectively.

The recovery of *B*. *anthracis* Ames and *B*. *subtilis* spores was measured across four independent experiments (2, 14, 28, and 56 days of exposure) on the four test surfaces with and without exposure to simulated sunlight; the overall trends of the data are presented in Figs 2–5. See also S1 Table for the tabulated spore recovery data. As stated previously, the response of the spores to UV-A/B exposure suggested a biphasic decay for both bacterial species, with a first phase of rapid inactivation (except for topsoil) in the first two days (initial slope), followed by a second phase at a generally lower rate of inactivation across the 56 day period (final slope fitted to data points using linear regression). The term “biphasic” was used by Sagripanti and Lytle [29] to describe similar trends in degradation kinetics for viruses exposed to UV radiation. This phenomenon has also been referred to as ‘tailing’, and has been suggested to occur due to microbe aggregation and clumping, light shielding, and/or from a resistant subpopulation of cells or viruses [4, 30–32]. Because of this overall observed biphasic decay, the detailed spore recovery results for the two phases are discussed separately below.

### Inoculation Levels and Initial Recovery of Spores

For all tests conducted, the number of CFU inoculated onto coupons (N_0_) ranged from 8.37 x 10^7^ to 1.11 x 10^8^ CFU for *B*. *anthracis* and from 7.83 x 10^7^to 1.04 x 10^8^ CFU for *B*. *subtilis*. The mean initial (i.e., from the 2-day experiment) recoveries of spores by species, UV exposure condition, and coupon material are presented in Table 2 in terms of N_2_/N_0_.

**Table 2:** Initial recovery of *B*. *anthracis* and *B*. *subtilis* spores (Average N_2_/N_0_ ± SD).

Material	UV-exposed			No UV		
	*Bacillus anthracis*	*Bacillus subtilis*	P-value	*B.a.*	*B.s.*	P-value
Glass	0.0002 ± 0.0001	0.0009 ± 0.0005	0.0190	0.821 ± 0.230	0.3110 ± 0.0165	0.0076
Bare Pine Wood	0.0164 ± 0.0052	0.0043 ± 0.0023	0.0015	0.0787 ± 0.0150	0.0319 ± 0.0045	0.0014
Unpainted Concrete	0.0657 ± 0.0072	0.0009 ± 0.0005	<0.0001	0.472 ± 0.213	0.0025 ± 0.0018	0.0078
Topsoil	3.68 ± 0.859	0.765 ± 0.154	0.0013	0.309 ± 0.112	2.55 ± 2.18*	0.082

p-values are for comparison between species

*This value not significantly different from 1.0

### Initial Recovery of Spores without UV Exposure

The recovery of spores at the two-day time point (N_2_/N_0_) from the non-UV exposed materials provides an estimation of the physical ability to recover spores as a function of material, since there would be no confounding effect of UV exposure or loss in viability due to extended time periods. With the exception of *B*. *subtilis* from topsoil, these recoveries ranged from approximately 0.25 to 82%, with the lowest recoveries (significantly different from other materials, with p-values < 0.05) obtained for bare pine wood and unpainted concrete. Although the highest initial recovery was obtained for *B*. *subtilis* from topsoil for the non-UV condition and was ostensibly greater than what was inoculated (N_2_/N_0_ = 2.55), statistical analysis determined this value was not significantly different from 1.0. In addition, examination of colony morphology and PCR tests confirmed that recovered CFUs were from the target organism and not native soil flora.

These initial spore recovery results without UV exposure are comparable to the recovery of *B*. *anthracis* and *B*. *subtilis* spores reported in the literature for positive controls used in decontamination studies [33–34]. In those decontamination studies, similar quantities of spores (i.e., approximately 1×10^8^ CFU) were inoculated onto the same types of materials as in the present study, and similar percent recoveries were obtained from the positive controls after drying overnight. As in the previous decontamination studies, initial recovery from bare pine wood and unpainted concrete was significantly less efficient than the recovery from glass, most likely due to the porosity and surface characteristics of the materials.

### Initial Recovery of Spores with UV Exposure

Except for *B*. *subtilis* on concrete (p-value = 0.109) and soil (p-value = 0.14), UV exposure significantly decreased the recovery of spores at the initial time point, with the effect of UV most pronounced on the non-porous material glass. The recovery of *B*. *anthracis* spores from glass (N_2_/N_0_ = 0.0002) at the initial time point was significantly less than from the other materials (all p-values < 0.002). The recovery of *B*. *subtilis* from both concrete and glass, i.e., N_2_/N_0_ = 0.0009, was significantly lower than for wood and soil. The highest recovery of spores at the two-day time point, with UV exposure, was from topsoil, and ranged from N_2_/N_0_ = 0.76 to 3.68 for the two species; more moderate recoveries were obtained from wood and concrete. Overall, these results suggest that pores in materials such as wood or concrete, or soil particles, may provide some shading from the UV exposure and diminish spore inactivation.

With respect to the recovery of *B*. *anthracis* spores from soil, the N_2_/N_0_ value was significantly greater than 1.0. Examination of colony morphology and PCR tests confirmed that recovered CFUs were from the target organism and not native soil flora. We also considered the possibility that nutrients in the topsoil allowed for the germination of spores, followed by population growth and then subsequent resporulation. However, we did not observe evidence of this “germination” hypothesis in an experiment performed in a separate, subsequent study evaluating soil decontamination for *B*. *anthracis* spores [35]. Other experimental errors may have caused this unexpected result, such as improper inoculation, errors in counting CFU, dilution plating, or PCR analysis.

We further note that all procedural and laboratory blanks met the criterion of no observed CFUs of the inoculated organism. Growth of native organisms, with colonies morphologically distinct from those of *B*. *anthracis* or *B*. *subtilis*, was observed from some blank topsoil coupons. However, CFU from native soil species were not observed when topsoil samples were spiked with the target organism, so no interference existed in terms of counting recovered spores.

### Second Phase of Spore Decay

The linear fits of the spore recovery data, i.e., N_t_/N_0_ (on log_10_ scale) versus time, are shown in Figs 2–5. The decay rates for the latter phase of the survival model (day 2 through day 56), i.e., the slopes of these linear regressions, are shown in Table 3. The data are presented in terms of fraction lost per day, as a function of material, species, and UV exposure regime.

**Table 3:** Decay rates for second phase of spore decay.

*Bacillus* species	Glass	Bare Pine Wood	Unpainted Concrete	Topsoil
**UV-exposed**				
B. anthracis	-0.084 ± 0.047	-0.065 ± 0.013	-0.063 ± 0.030	**-0.017 ± 0.004**
B. subtilis	**-0.117 ± 0.020**	-0.043 ± 0.023	-0.056 ± 0.020	**-0.003 ± 0.008**
P-value < 0.05?	no	no	no	yes
**Unexposed**				
B. anthracis	**0.070 ± 0.014**	-0.035 ± 0.013	-0.023 ± 0.011	**0.026 ± 0.007**
B. subtilis	**-0.038 ± 0.014** ^**α**^	-0.014 ± 0.009	-0.011 ± 0.009	-0.018 ± 0.019
P-value < 0.05?	yes	no	no	yes

Data are presented as the mean decay rates (day^-1^) ± 95% confidence interval. Numerical results in bold indicate that a decay rate for a particular material/species combination is significantly different at the 5% confidence level from the other materials. Whether a P-value is less than 0.05 is listed for a comparison within a material and across the two species.

^α^For *B*. *subtilis*, a significant difference is present between glass and unpainted concrete/bare pine wood, but not between glass and topsoil.

Although there were a few exceptions, decay rates in the second phase were significantly highest for glass and lowest for soil, and ranged from a high of -0.117 (glass/*subtilis*/UV)—to a low of -0.003/day (topsoil/*subtilis*/UV). There were no significant differences in the decay rates between concrete and wood, for any species/UV light combination. All the decay rates shown in Table 3 are significantly different from zero, except for *B*. *subtilis* in topsoil for both the UV and no UV exposure conditions.

We also note that the decay rate for *B*. *anthracis* in topsoil with no UV is actually an increase, an artifact from having a slightly lower recovery at the two-day time point compared to the recovery at 56 days. The decay rate for *B*. *anthracis* in topsoil samples that were exposed to UV averaged -0.017/day, which is biased high due to the abnormally high recovery at N_2_.

While the rates of decay in spore recovery for the second phase were generally higher for the UV-exposed materials, actual significant differences due to the UV exposure condition depended on the material and species. For *B*. *subtilis*, significantly higher decay occurred under the UV treatment for both glass and concrete, but there was no significant effect of UV exposure on either wood or soil. Further, the decay rate of *B*. *anthracis* on glass, concrete, or wood was not significantly different when comparing between the UV and no-UV exposure conditions. The cases for lack of any significant effect of UV during the second decay phase is presumably due to remaining viable spores (after the rapid inactivation phase) being shielded via the pores in the material (wood, concrete) and/or shielded by the top layers of spores (already inactivated) that may have aggregated (clumping due to inoculation process) on glass. This shielding due to material effects or aggregation of spores on the material is a more realistic reflection of what would occur in nature [4] (e.g., a deliberate release of spores outdoors), as opposed to studies in which monolayers of spores are applied to non-porous materials [5, 6]. While we did not microscopically examine the spore deposition onto the materials, we expect that there would be some aggregation of spores, since we made no attempt to deposit spores as monolayers. Indeed, in a study using similar spore suspension inoculation methods onto materials, the authors did report spore clumping and aggregation [36].

### Decay of Viable Spore Recovery as a Function of UV Dose

**Fig 6:**
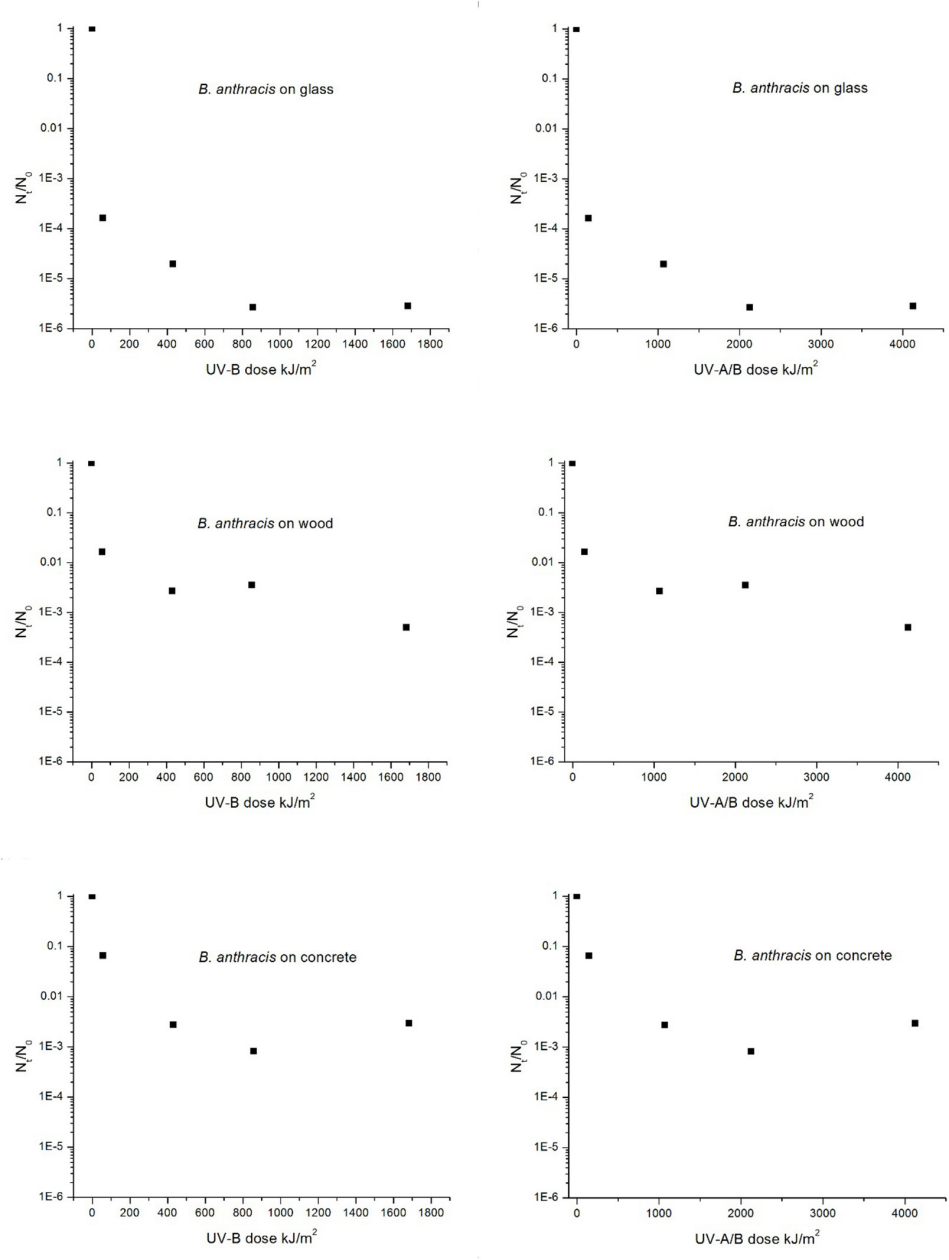
Decay of *B*. *anthracis* spores as a function of UV dose (kJ/m^2^), species, and material. Dose values are shown for UV-B and UV-A/B and were determined based on measured UV intensities and exposure time.

**Fig 7:**
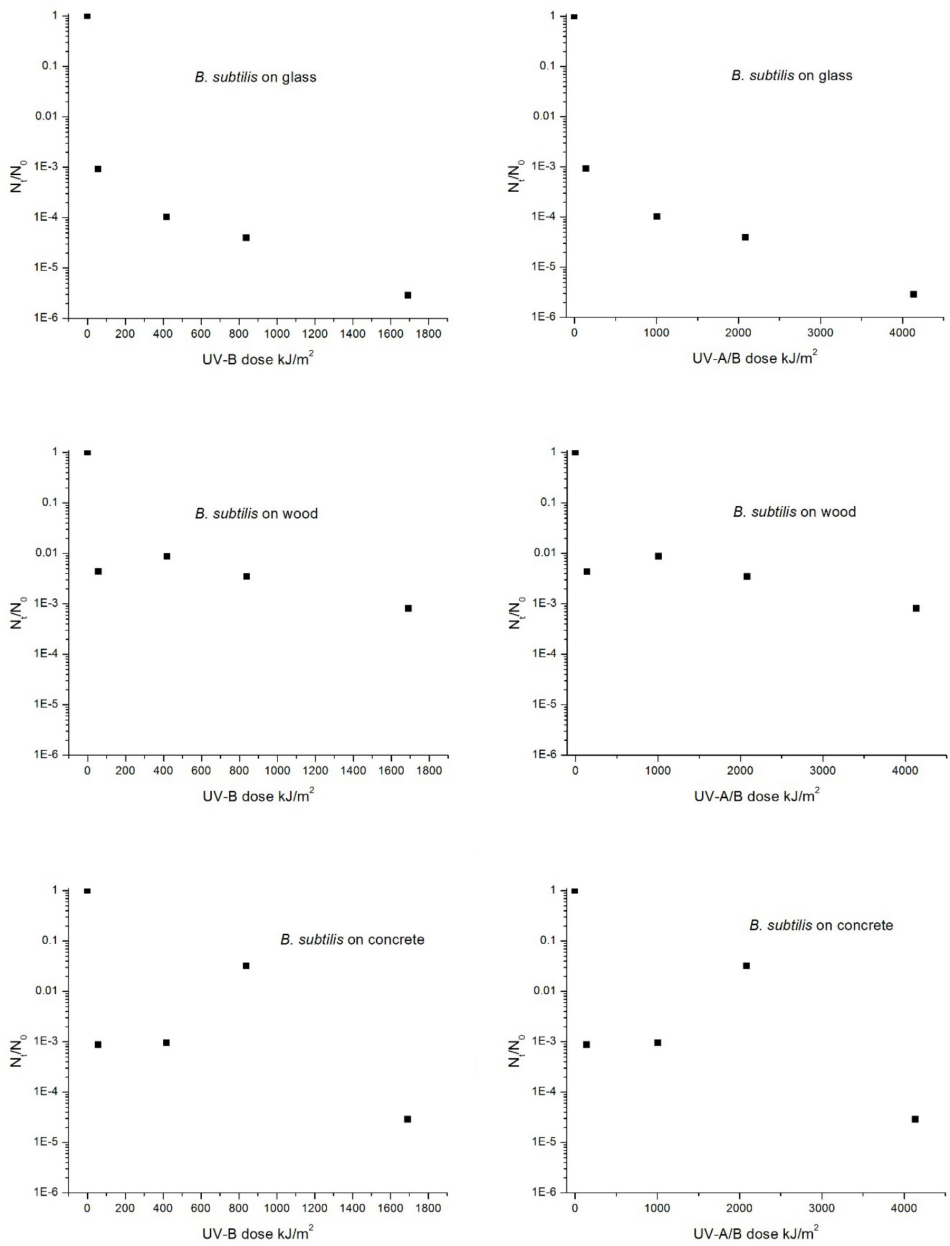
Decay of *B*. *subtilis* spores as a function of UV dose (kJ/m^2^), species, and material. Dose values are shown for UV-B and UV-A/B and were determined based on measured UV intensities and exposure time.

The initial and second phase decay rates for both species have also been assessed in terms of UV dose (kJ/m^2^) for the glass, wood, and concrete materials; refer to Figs 6 and 7 for *B*. *anthracis* and *B*. *subtilis*, respectively. Although we were unable to find any data in the literature with regard to the kinetics of *B*. *anthracis* spore inactivation upon exposure to components of solar radiation (UV-A and/or UV-B), several studies have provided kinetic data for *B*. *subtilis* [5,6,12–14]. These studies provided the dose required to reduce the initial population by 90%, referred to as the LD_90_, or also the D-value if monophasic, and thus are comparable to the LD_90_ values calculated based on our initial recovery data. Additionally, these studies typically used microscope or quartz slides as the substrate material for deposition of spores, and consequently their data are most comparable to our results for glass.

For the present study, the LD_90_ for UV-B for the decay of the *B*. *subtilis* population on glass was determined to be 19 kJ/m^2^, and for *B*. *anthracis*, the UV-B LD_90_ was determined to be 15 kJ/m^2^. These data are comparable to the *B*. *subtilis* UV-B LD_90_ values reported by Xue et al. [14], Riesenman et al. [12], and Slieman et al. [13], which ranged from 22 to 66 kJ/m^2^. If we include the UV-A portion of UV dose for our study, the LD_90_ values for UV-A/B exposure for spores on glass were determined to be 48 and 39 kJ/m^2^ for *B*. *subtilis* and *B*. *anthracis* respectively. This compares to the UV-A/B LD_90_ values reported in the literature for *B*. *subtilis*, which ranged from 7.8 to 101.5 kJ/m^2^ [5,6,12,14–15]. We acknowledge that comparing biological agent decay data from the literature can be challenging, due to the myriad differences in experimental methods and materials. For example, differences in *B*. *subtilis* strains, inoculum levels, spore preparation methods, range in UV wavelengths used, intensity and spectrum of UV sources, different methods for placement of spores onto materials (e.g., use of monolayers), all could potentially contribute to differences in results. In addition, we acknowledge our LD_90_ values were determined using only the data for recovery at day 2 and that additional data of spore recovery at more frequent and earlier time intervals would be more appropriate for determining LD_90_ values.

While the overwhelming majority of decontamination and related studies similar to the present study deposit spores on to materials via a liquid suspension, we acknowledge that aerosol deposition of spores onto materials is an emerging technique that may better represent how *B*. *anthracis* spores would accumulate on materials in an actual scenario. Further, it is likely that the inoculation method could have a significant effect on inactivation rates. This was demonstrated in a study investigating chemical decontaminants, in which Ryan et al. [37] showed that inactivation rates were indeed affected by the spore inoculation method.

### Recovery of Spores at 56 Days

The results for the recovery of spores at the longest elapsed time tested are presented in Table 4 to provide an overall perspective on spore persistence. The data show that the recovery of viable spores is diminished when exposed to the simulated sunlight on all materials except topsoil. The exposure to UV-A/B resulted in approximately a 1 to 2 log reduction on glass, wood and concrete, when N_2_/N_0_ (Table 2) is compared to N_56_/N_0_. On glass in particular, there was an overall 6 log decrease in recovery at the 56-day time point (comparing N_0_ to N_56_).

**Table 4:** Recovery of spores at 56 days (N_56_/N_0_ ± SD).

Material	UV-exposed		No UV	
	*Bacillus anthracis*	*Bacillus subtilis*	*B.a.*	*B.s.*
Glass	2.83 x 10^−06^ ± 6.11 x 10^−06^	2.890 x 10^−06^ ± 3.19 x 10^−06^	0.0155 ± 0.0071	0.0775 ± 0.0231
Bare Pine Wood	0.0005 ± 0.0004	0.0008 ± 0.0005	0.0093 ± 0.0024	0.0138 ± 0.0027
Unpainted Concrete	0.0030 ± 0.0049	2.86 x 10^^-05^ ± 5.95 x 10^^-06^	0.0386 ± 0.0095	0.0040 ± 0.0006
Topsoil	0.522 ± 0.0939	0.5040 ± 0.1000	0.849 ± 0.316	0.702 ± 0.0760

Without exposure to UV, we observed an approximately ten-fold (i.e., 1 log) reduction in the recovery of both species on glass, concrete, and wood, between the initial and final time points (again, comparing N_2_/N_0_ to N_56_/N_0_). Although there are limited data with which to compare, this result is analogous to a study conducted by Dietz et al. [38] who reported a 70–90% reduction in the recovery of *B*. *anthracis* spores from a few nonporous materials after 100 days, when the materials were kept at room temperature with no UV exposure.

For topsoil, there was minimal loss in viable spores over the 56-day test period, independent of species or UV exposure condition. This observation is consistent with anecdotal evidence reported in the literature, in which spores have been shown to remain viable or present for decades in soil. See, for example, Manchee et al. [39].

### Comparing Results for *B*. *subtilis* and *B*. *anthracis*

Significantly lower initial recoveries of spores were observed for *B*. *subtilis* compared to *B*. *anthracis*, for both UV exposure conditions and for nearly all materials (Table 2). For the non-UV exposed materials, the generally lower recovery of *B*. *subtilis* is consistent with the results obtained from positive controls used in decontamination studies [33, 34]. With regard to comparing decay rates of the two species (Table 3), the rate of decay for *B*. *subtilis* was either statistically equivalent or was significantly less than that of *B*. *anthracis*. Thus while the initial recovery of spores from materials may generally be lower for *B*. *subtilis* (which may be due to a physical phenomenon), the resistance to inactivation over time on materials is generally higher for *B*. *subtilis*, suggesting that *B*. *subtilis* may be an appropriately conservative surrogate for *B*. *anthracis* for environmental persistence studies.

In summary, the results of the present study assist in elucidating the conditions and estimated time for which spores of *B*. *anthracis* may remain viable in the environment. The results suggest that natural attenuation of *B*. *anthracis* spores in the environment may have limited value as a potential remediation option following an accidental or intentional release, except for spores found on clean, nonporous materials, and exposed to ample sunlight.

## Disclaimer

The U.S. Environmental Protection Agency through its Office of Research and Development funded and directed the research described herein under GS23F0011L-3 with Battelle. It has been subjected to the Agency’s review and has been approved for publication. Note that approval does not signify that the contents necessarily reflect the views of the Agency. Mention of trade names, products, or services does not convey official EPA approval, endorsement, or recommendation.

## Supporting Information

S1 Table Inoculum and recovery data for each test of the entire study. (XLSX)
